# Plastic plumage colouration in response to experimental humidity supports Gloger’s rule

**DOI:** 10.1038/s41598-023-28090-5

**Published:** 2023-01-16

**Authors:** Isabel López-Rull, Concepción Salaberría, Juan Antonio Fargallo

**Affiliations:** 1 Departamento Biología y Geología, Física y Química Inorgánica, Área de Biodiversidad y Conservación, Universidad Rey Juan Carlos, C/Tulipán s/n., 28933 Móstoles, Madrid, Spain; 2 Departamento de Ecología Evolutiva, Museo Nacional de Ciencias Naturales-CSIC, José Gutiérrez Abascal 2, 28006 Madrid, Spain

**Keywords:** Ecology, Evolution, Zoology, Ecology

## Abstract

Knowing how animals adapt their phenotype to local temperature and humidity is key to understanding not only ecogeographical rules, but also how species will manage climate change, as current models predict changes in global patterns of temperature and precipitation. In endotherms, colour adaptations in response to climate have been under investigated, and their acclimatization-the individual capacity to reversibly adjust phenotype in response to different environments-is unknown. Geographic trends can provide clues about abiotic variables involved in colouration, as postulated by Gloger’s rule, which predicts darker individuals in warm and humid regions. We tested whether house sparrows (*Passer domesticus*) can adjust colouration when faced with varying humidity conditions. We exposed birds to either a dry (humidity 45%) or a wet environment (70%) six months before their moult, and measured colouration in newly developed feathers in five parts of the body (bib, crown, crown stripe, belly and rump). As predicted by Gloger’s rule, birds in wet conditions developed darker (bib and belly) and larger (bib) melanised plumage patches, than birds in dry conditions. Our result provides the first unequivocal evidence that the ability of individual birds to adjust their colouration may be a potential adaptation to climatic changes in endotherms.

## Introduction

Phenotypic plasticity is the ability of a single genotype to express different phenotypes depending on environmental conditions^1^. One type of phenotypic plasticity is acclimatization, which refers to reversible, temporary, and repeatable phenotypic adjustments within a single individual in response to changing environmental conditions^2^. Such an ability is an important component of fitness for organisms that face variable environments^3^. Understanding how acclimatization generates functional variability has become increasingly important in the context of climate change, as current models predict changes in global patterns of temperature and precipitation^4,5^.

Animal colouration is one of the most diverse phenotypic traits and is known to vary along climatic gradients, as described by two ecogeographical rules: Bogert and Gloger’s rules^6,7^*.* Whereas both rules account for geographical variation in colour due to the differential deposition of melanic pigments, their predictions contradict each other: Bogert´s rule, initially proposed for ectothermic animals, predicts darker individuals in cold environments, based on the thermal advantages of darker phenotypes which generally heat more and faster than paler ones^6,8^. In contrast, Gloger’s rule, initially proposed for endothermic animals, predicts darker individuals in warm and humid environments^7^. Although the mechanism behind Gloger’s rule is not clear, thermal regulation may not be the only explanation as endotherms mainly rely on internal metabolic processes to maintain their body temperature. Currently, selection for more cryptic-darker phenotypes and increased protection against keratinolytic bacteria by melanin deposition in warm humid environments are two main explanations when this biogeographical pattern is found^9^.

However, colouration has several other functions, from cryptic camouflage to conspicuous visual signals. So, explanations for its variation are far from simple and individual colouration does not necessarily follow the patterns one would expect from Gloger’s rule^8–10^. This is particularly true in birds, as this taxon shows the most diverse colours among animals. Recently, it has been shown that patterns predicted by Gloger’s rule are mainly associated with variation in humidity rather than temperature^9^. Further, the effects of temperature on colouration have been found to oppose Gloger’s rule predictions, namely that species/individuals with darker plumage are often found in colder rather than in warmer regions^11–16^. Therefore, it has become necessary to disentangle the effects of temperature and humidity on melanin deposition.

A main point in understanding the underlaying mechanism of Gloger’s rule is to test the ability of individuals to adjust their colouration to fit their environment. Knowing how populations adapt their colour to local temperature and humidity conditions is key to understanding colour-related biogeographical patterns. The observed colouration in a population may be the product of microevolution (gene frequency) or of phenotypic plasticity, including acclimatization. Unlike ectotherms, colour adaptations in endotherms in response to climatic changes have been under investigated, and their phenotypic plasticity associated with humidity and temperature acclimatization is currently unknown. It has been experimentally shown that environmental growth conditions can vary the melanic colouration in nestlings of house sparrows *Passer domesticus* and common kestrels *Falco tinnunculus*^17,18^. Also, a recent work with nestling griffon vultures *Gyps fulvus*^19^ suggested birds have the capacity to respond to local sunlight conditions by adjusting melanin deposition in growing feathers, which influences fitness. Yet, experimental support for such capacity is lacking, and to our knowledge, only two outdated works suggest the idea of humidity and temperature may promote darker colours, one conducted in birds and one in mammals^20,21^. Such relevant findings urge confirmation with a solid experimental design.

The purpose of our study was to test phenotypic plasticity in melanin colouration in endotherms in relation to variation in climatic conditions. Specifically, we tested whether birds may acclimatize their plumage colouration after moult in response to varying conditions of humidity, since humidity appears to be the core driver behind Gloger’s rule. We used the house sparrow as a model species because its plumage is coloured through melanin deposition and because it has been reported that introduced house sparrow populations have apparently rapidly changed their coloration to local climatic variation^22^ suggesting a role for phenotypic plasticity. We exposed house sparrows to either a dry or a humid environment six months before their moult, and measured plumage colouration in newly developed feathers. If humidity promotes a higher deposition of melanin pigments, as assumed by Gloger’s rule, birds housed in the humid environment should be darker than birds housed in the dry environment.

## Material and methods

In February–March 2021, 54 adult male house sparrows were captured with mist nests from a wild population at the ZOO Aquarium in Madrid. All individuals were marked with metallic rings. After capture, birds were transported to the Foundation for Research in Ethology and Biodiversity (FIEB Foundation) and confined randomly in 4 indoor aviaries (3.3 m long × 2.7 m wide × 2.1 m tall; each aviary containing 13–14 birds) that were built with insulating material and had climate-controlled housing. Birds in the aviary were maintained at ambient temperature/humidity and under a natural photoperiod. All aviaries had a north-facing window (1 m × 1 m) and contained plants and shrubbery to simulate a natural environment. After one month of habituation to captivity, the experiment was performed. During both habituation and experimental periods, birds were provided *ad libitum* with water, a commercial mixture of seeds for canaries (VINCI) and cuttlefish bones to meet calcium needs (see^23^, for a similar protocol of feeding for captive wild sparrows). To ensure room ventilation, aviary windows were opened twice a day (at 9:00 and at 18:00 h) for one hour. Aviaries were checked daily, and the feeders and drinkers were cleaned every day. A veterinary revision was carried out four days a week.

### Individual biometry and colouration

Measurements of tarsus length, body mass and plumage colouration were taken at the beginning of the experiment. Tarsus and body mass measurements were taken with a digital calliper and balance, respectively. Plumage colouration was measured in 5 different parts of the body (the black bib, the grey crown, the rufous lateral crown stripe, the pale grey belly, and the grey rump) using a MINOLTA spectrophotometer (CM26d) which measures the reflectance from 360 to 700 nm in intervals of 10 nm. Reference calibrations were performed against zero and a white standard tablet associated with the apparatus according to the instructions provided by the maker. Reflectance spectra were automatically produced as means of 3 sequential measurements. The SPECTRAMAGIC software (MINOLTA) was used to analyse spectra. Total Reflectance was obtained as the summed reflectance at each 10-nm interval from 360 to 700 nm. Total Reflectance is a measure of the total amount of light reflected by the feathers^24^ thus as total reflectance increases, plumage tends to get lighter (^25^, hereafter we will refer to lightness). Also, to measure the area of the black chest bib, digital photographs from the ventral side of the bird were taken while the bird was held at an angle of 90° between the objective of the camera (Canon EOS 77D) and the surface of the body. Photographs were taken under a sunshade in the morning. Images were imported into Adobe Photoshop CS6, where the size of the bib was measured by selecting the total black area on throat/chest and converting pixels to cm^2^^26^. The repeatability^27^ of bib size based on 20 males measured twice was high (r = 0.97, *p* < 0.0001).

### Experimental design

To test the effect of ambient humidity on plumage colouration, on 14 April 2021 we exposed house sparrows to either a “Dry” (relative humidity 45%; N = 27) or a “Wet” (relative humidity 70%; N = 27) environment at 20ºC. The birds were housed in each of the four aviaries (two for the wet treatment and two for the dry treatment) as they were being released one by one from the collectors to maintain a similar number of animals in each aviary after each release. In this way we ensured a random accommodation of individuals in relation to size, body mass and starting plumage colouration among groups (General Lineal Model GLM, all *p* > 0.16, n = 54). Constant temperature was achieved using air heaters on thermostats in each aviary. Humidity conditions were controlled either by humidifiers in the wet aviaries, and by sorption dehumidifiers in the dry aviaries, both with humidity sensors to precisely maintain humidity. Two thermohydrometers located inside each aviary allowed us to monitored temperature and humidity daily (at 8:00 and 19:00 h). The relative humidity values we chose are within the natural range where the birds were captured from (the average relative humidity in Madrid is 57%, ranging from 38 to 74%; Information prepared by the State Meteorological Agency AEMET). The duration of the experimental treatment was 6 months. In the house sparrow, the moulting period takes place at the time our experiment was conducted (i.e., July–September^28^), so, after the end of the treatment (14 October 2021) all birds had developed a new plumage. On 14 October 2021, body mass and plumage colouration were measured. When the experiment concluded, birds were released at their capture place.

To analyse the effect of ambient humidity on plumage colouration we used General Lineal Models (GLM) that included colour traits after treatment as the dependent variables, treatment as a fixed factor and body mass as a covariate. The initial colour (or size) of each plumage trait was also included as a covariate to increase the precision of the estimates^29^. In the case of bib size, tarsus length was also included in the model as a covariate to control for allometric effects. All colour variables, except bib size, were log transformed and residuals from all models were normally distributed. Statistical analyses were performed with STATISTICA software. The standardized values of the plumage colour variables are shown in Table S1 (Supplementary Material). Pictures from the bib size before and after the treatment are shown in Figure S1 (Supplementary Material).

### Ethical statement

All methods of this research were carried out in accordance with the ethical guidelines proposed for the Spanish Royal Decree 53/2013 which establishes the rules applicable for the protection of animals used in experiments and scientific research. All experimental protocols were approved by the Research Ethics Committee of the Rey Juan Carlos University, as the organism authorized by the Comunidad Autónoma de Madrid for evaluation of projects based on what is stated in RD 53/2013 (Ref:1011202020220). All methods were carried out in accordance with ARRIVE guidelines. Permissions to conduct the study were granted by the Comunidad Autónoma de Madrid and Junta de Castilla la Mancha. The experimental work was supervised by the veterinary staff from FIEB.

During the experiment 7 males from the wet treatment died (4 from one aviary and 3 from the other one). The corpses were analysed by the FIEB veterinarians who determined a possible mycosis as the cause of death. Prior to the date of death, no birds (except one) showed apparent signs of disease in their routine health assessments done by the veterinarians (birds were checked four days a week). The only bird with disease signs was observed perched in the ground showing no interest in food or water. Immediately after this observation the experiment was finished for this bird and it was isolated in an individual cage at room temperature and “normal” humidity conditions. The bird died within a few hours, before the veterinarians could administrate any antifungal medication. No deaths were recorded in the dry treatment. Thus, final samples sizes are 27 individuals in the dry treatment and 20 individuals in the wet treatment.

As this is the first work in which humidity was manipulated in these ranges-which are within the natural variation of humidity faced by house sparrows in their original population-we could not have foreseen this outcome. Yet, because mortality only occurred in the high humidity group, and because the suspected cause of death is likely linked to the treatment, we consider this information very important for future researchers attempting similar experiments.

## Results

**Figure 1:**
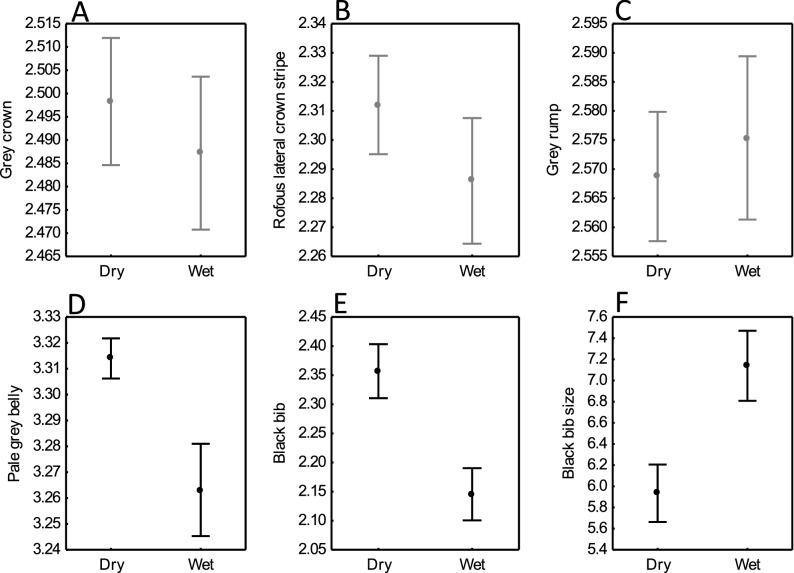
House sparrow plumage colouration (log-transformed) and black bib size differences between dry and wet treatments in the house sparrow. Whiskers represents mean ± standard deviation. Significant effects are shown in black while non-significant effects are shown in grey.

Controlling for initial colouration, no differences between groups were found after exposure to the humidity treatments in the lightness of the grey crown, the rufous lateral crown stripe or the grey rump (Table 1; Fig. 1). However, birds from the wet treatment were significantly darker in the pale grey belly and the black bib and, also had larger bibs than those from the dry treatment (Table 1; Fig. 1). No correlation was observed between bib size and body size, measured as tarsus length (GLM, *p* = 0.30).

**Table 1:** Results of the GLMs for plumage colouration and the black bib size of house sparrows as a function of treatment (dry (0) *vs*. wet (1)).

Term	Estimate	SE	F	*p*	Lower 95% CI	Upper 95% CI
Grey crown colouration						
Treatment	− 0.001	0.011	0.010	0.923	− 0.024	0.021
Body mass	− 0.010	0.006	3.010	0.090	− 0.022	0.002
Initial colour	0.012	0.025	0.239	0.627	− 0.037	0.061
Rufous lateral crown stripe colouration						
Treatment	0.016	0.015	1.588	0.288	− 0.014	0.045
Body mass	0.004	0.008	0.205	0.653	− 0.012	0.019
Initial colour	− 0.016	0.032	0.244	0.610	− 0.080	0.048
Grey rump colouration						
Treatment	− 0.006	0.010	0.365	0.549	− 0.025	0.014
Body mass	− 0.003	0.005	0.382	0.540	− 0.014	0.008
Initial colour	− 0.021	0.019	1.213	0.277	− 0.059	0.017
Grey belly colouration						
Treatment	0.028	0.009	8.941	**0.005**	0.009	0.046
Body mass	0.008	0.005	2.776	0.103	− 0.002	0.018
Initial colour	− 0.032	0.019	2.948	0.093	− 0.070	0.006
Black bib colouration						
Treatment	0.113	0.036	9.949	**0.003**	0.041	0.185
Body mass	0.011	0.020	0.320	0.574	− 0.028	0.051
Initial colour	− 0.023	0.083	0.077	0.783	− 0.190	0.144
Black bib size						
Treatment	− 0.717	0.224	10.289	**0.003**	1.168	− 0.266
Body mass	− 0.189	0.119	2.519	0.120	− 0.428	0.051
Initial colour	0.067	0.129	0.269	0.606	− 0.193	0.327

Effects of body mass and colour at the beginning of the experiment the are also shown. n = 27 (dry group) and n = 20 (wet group) and d. f. = 43. Bold type represents statistically significant effects of treatment in plumage traits.

Body mass at the end of the experiment was found to differ between groups. Males from the wet treatment were heavier than males from the dry treatment (dry: 24.89 ± 1.62 g; wet: 26.15 ± 2.00 g; F_1,45_ = 5.74, *p* = 0.02). Although body mass had no significant effect on the grey crown, the rufous lateral crown stripe, the grey rump, the black bib or the bib size (Table 1), it was marginally correlated with belly colouration (Table 1), so we decided not to remove it from the analyses.

## Discussion

We found evidence of phenotypic plasticity in melanin-based colouration in endotherms dependent on climatic conditions: adult sparrows confined for six months to wet aviaries developed darker and larger plumage traits than those confined to dry aviaries. The direction of such acclimatization in plumage colouration in response to humidity (darker when wet) is consistent with both the pioneering work of Beebe^20^ and the predictions of Gloger’s rule^7^.

In relation to the former, Beebe found that individuals of three different species that were kept in indoor aviaries in wet conditions developed darker feathers after 2–3 years of exposure to humidity, when compared to those that remained outdoors^20^. Despite the small sample sizes of Beebe´s experiments (*Hylocichla mustelina* (n = 2)*, Zonotrichia albicollis* (n = 1) *and Columbina inca* (n = 1; sample size is not clear and data is only available for one individual) and the lack of real controls, his study was innovative in suggesting plasticity in plumage colouration. Since then, the ability of birds to adjust their plumage colouration in response to climatic variables had only been tested twice, first by Fargallo et al.^19^ showing the potential role of solar exposure in nestling feathers colouration in vultures, and now this study in captive adult sparrows, in which we provide the first unequivocal evidence of colour acclimatization in response to humidity.

In relation to the predictions of Gloger’s rule, this study supports the idea that darker individuals are expected in humid conditions due to the increased deposition of melanin pigments^7^. Sparrows in the wet treatment developed not only darker (belly and bib) but also larger traits (bib) than those in the dry environment, indicating more melanin deposition, as the bib black colouration is produced by melanic pigments^30^. Melanin pigments are grouped in two main chemical variants: eumelanin and pheomelanin which in different combinations produce a broad range of black, brown, gray and reddish colors^31^. To date, it is not really understood how the final coloration derives from both pigments and physical disposition and shape of melanosomes influences feather coloration^32^, yet many authors have assumed that black/gray is eumelanin dependent; and yellow, brown, and reddish are pheomelanin dependent, considering the most prevalent pigment in each case (e.g. ^33–38^.). Gloger’s rule, can be defined in a simple or a complex way. In its simple version, it, states that endothermic animals are predicted to be darker in warmer and humid areas due to the increased deposition of melanin pigments. In a more complex version, climatic variables are supposed to correlate in different ways with the deposition of the two melanin pigments: whereas the deposition of eumelanins is predicted to increase with humidity and decrease only at extreme low temperatures, the deposition of pheomelanin is higher in dry and warm regions and decrease rapidly with lower temperatures. Currently, however, we know that pale and dark coloration does not necessarily have to be associated with the type of melanin pigment. For example, brown-reddish coloration of feathers has more eu- than pheomelanin^37,38^, the degree of reddishness is associated to the concentration of both pheo- and eumelanin pigments^37^, grey coloration has a higher eu/pheo ratio than the black coloration^18^, and darkness in feathers is explained by the total amount of melanin rather than by the concentration of one of the two pigments^19,38^. So, in the absence data quantifying the deposition of both eu- and pheo-melanin the evidence from the rule refers to the simple version. Interestingly, among all plumage traits measured, darker colouration in the black bib is particularly relevant since it is known that male house sparrows with larger bibs are dominant in aggressive interactions, have better body condition and a higher lifetime reproductive success^30^. Thus, such acclimatization of endotherms through the modification of colouration in a socially selected trait may affect their sexual and non-sexual interactions, and ultimately their fitness.

The physiological mechanism(s) underlying the change in plumage colouration in response to humidity is unknown. The types and amounts of melanins produced by melanocytes during melanogenesis are genetically and environmentally controlled^39–41^. In birds, melanogenesis is influenced by a variety of intrinsic and extrinsic factors such as hormonal changes, exposure to UV light, food quality and nutrition, or parasite infestation^18,42–46^. Whether these stimuli may be affected by humidity and thus influence the different pathways in melanogenesis warrant further investigation.

Recently, it has been shown that the increase in ambient humidity accelerates melanin photodegradation^47^. This provides a plausible explanation for our finding that birds confined to a humid environment produce more melanin, as this may be to counteract melanin degradation in humid regions. In fact, the idea behind Gloger’s rule that dark colouration confers selective advantages can be explained by different selective forces that are not necessarily mutually exclusive. For example, individuals may be darker in wetter habitats because melanin pigments play a role in thermoregulation and birds with heavily pigmented feathers repel water better, allowing the body to dry more rapidly^48–50^. Also, darker animals in humid environments could benefit from camouflage, since in more humid regions, the soil is darker (rich in organic matter), and the vegetation is denser, which limits the entry of light and produces a dark and shadowy environment^51^. Parasites and pathogens living in humid habitats may also exert a selective pressure, as darker birds have been shown to be more resistant to them due to increased melanin pigments that protect the integument against ectoparasites^52^ and feather degrading bacteria^52–54^. In fact, there is evidence that the melanin deposited in the feathers confers the feathers strength and makes them more resistant to feather-degrading bacteria^52,54^. Finally, evolution might favour melanic coloured traits to be phenotypically integrated with other traits, such as immunity and hormonal profiles via pleiotropy, genetic or functional correlations^38,55,56^. Hence, if the prevalence of parasites and pathogens varies along humidity gradients, selection would favour stronger immune responses in warm-humid zones^57^ which darker individuals may possess. Whatever the mechanism, it is known that that house sparrow coloration rapidly adapts to local climatic conditions after colonization^22^. So, the results of our experiment would suggest that a component for such adaptation might be the plasticity observed in this study, which is an important but ill-understood component promoting rapid phenotypic changes.

Differences in body mass did not affect plumage colouration. Yet, an interesting result from our study is the fact that that body mass differed between treatments: birds in the wet treatment were heavier than those in the dry treatment. Differences in body mass in relation to treatment is an important result since it adds experimental evidence to a recent work that found that increased humidity was associated with increased body condition in 24 of 46 passerine bird species^58^. The proximate explanation for this association may be that humidity can impact heat retention and fuel composition which can, in turn, impact lean mass^59,60^. 

In summary, our results show birds have the capacity to respond to local conditions of humidity by changing plumage colouration due via adjustments in melanin deposition between moults. Both the proximate and the ultimate causes of such acclimatization in plumage colour traits in response to humidity, as well as its ecological implications remain unsolved. Yet we provide the first unequivocal evidence that the ability to adjust individual colouration may be a potential adaptation to climatic changes in endotherms.

## Supplementary Information

Supplementary Information.

## Data Availability

The raw data supporting the conclusion of this article will be made available by the authors, without undue reservation. ILR should be contacted if someone wants to request the data from this study. The datasets generated and/or analysed during the current study are stored in the following data repository: http://repositories.biodiversos.org/Lopez-Rull_Isabel/Scientific%20Reports_Plastic%20colouration%20and%20%20humidity%20in%20birds/.
