# Supplementary material for: Plastic plumage colouration in response to experimental humidity supports Gloger’s rule
Source: Sci Rep. 2023 Jan 16;13:858. doi: 10.1038/s41598-023-28090-5 (PMC9842646; doi:10.1038/s41598-023-28090-5)

**Supplementary Material**

**Table TS1:** Between-treatment differences in standardised plumage colour variables. Variables showing significant differences are shown in bold.

|  | **All groups** | |  | **Dry** | |  | **Humid** | |  | **Difference** |  | ***S.D.*** |
| --- | --- | --- | --- | --- | --- | --- | --- | --- | --- | --- | --- | --- |
|  | mean | *S.D.* |  | mean | *S.D*. |  | mean | *S.D.* |  |  |  |  |
| Grey crown | 2.49 | 0.07 |  | 2.50 | 0.07 |  | 2.49 | 0.07 |  | 0.01 |  | 0.16 |
| Rufous lateral crown stripe | 2.30 | 0.09 |  | 2.31 | 0.09 |  | 2.29 | 0.10 |  | 0.03 |  | 0.28 |
| Grey rump | 2.57 | 0.06 |  | 2.58 | 0.04 |  | 2.58 | 0.06 |  | 0.00 |  | 0.06 |
| **Pale grey belly** | 3.29 | 0.06 |  | 3.31 | 0.04 |  | 3.26 | 0.08 |  | 0.05 |  | 0.79 |
| **Black bib** | 2.27 | 0.25 |  | 2.36 | 0.24 |  | 2.15 | 0.20 |  | 0.21 |  | 0.86 |
| **Bib size** | 6.45 | 1.55 |  | 5.93 | 1.41 |  | 7.14 | 1.48 |  | 1.21 |  | 0.78 |

**Figure FS1: Photographs randomly chosen from 6 individuals (3 from the dry treatment and 3 from the wet treatment) before and after treatment.**


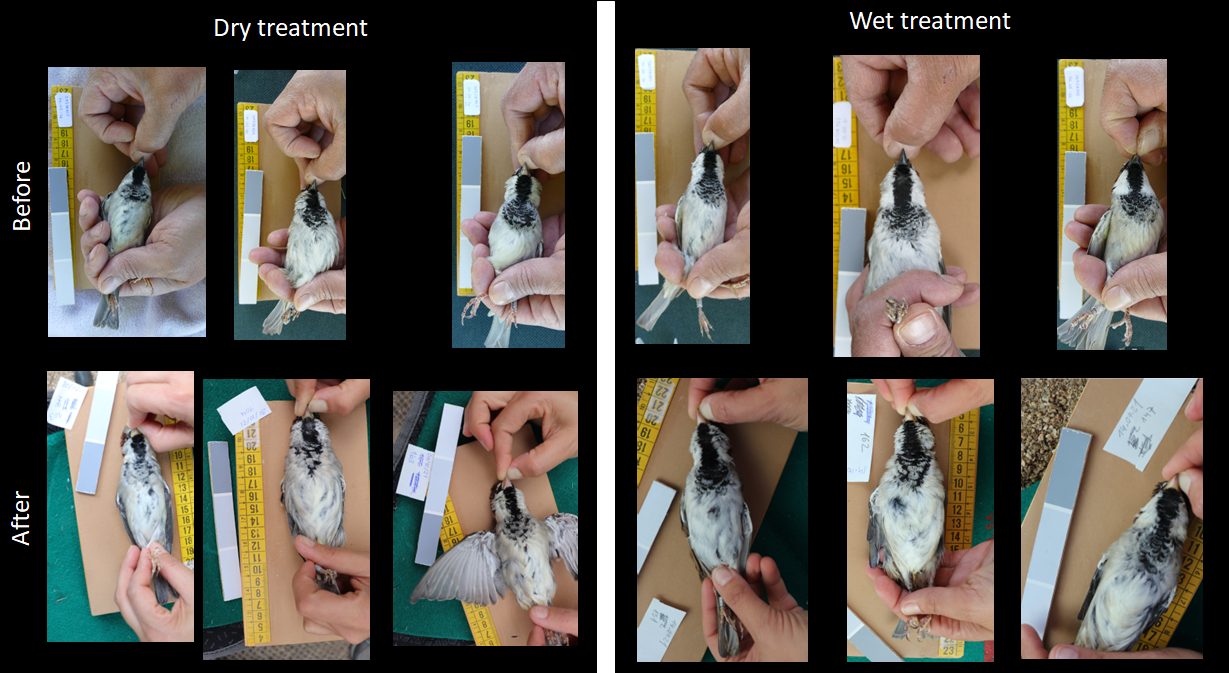

Supplement: Supplementary file 1 — Supplementary Information. [file 41598_2023_28090_MOESM1_ESM.docx]
